# Long-term survival following total pancreatectomy and superior mesenteric-portal vein resection for pancreatic ductal adenocarcinoma: A case report

**DOI:** 10.3892/ol.2014.2628

**Published:** 2014-10-22

**Authors:** HAN-XING TONG, LEI ZHANG, YE-FEI RONG, DAN-SONG WANG, TIAN TAO KUANG, XUE-FENG XU, WEN-HUI LOU, DA-YONG JIN

**Affiliations:** Pancreatic Cancer Group, Department of General Surgery, Zhongshan Hospital, Fudan University, Shanghai 200032, P.R. China

**Keywords:** pancreatic ductal adenocarcinoma, superior mesenteric-portal vein resection, long-term survival

## Abstract

Pancreatic ductal adenocarcinoma (PDAC) is an aggressive cancer with few therapeutic options. At present, surgical resection remains the only potential curative treatment for PDAC. However, only 15–20% of patients with PDAC are eligible for lesion resection. Total pancreatectomy (TP) and superior mesenteric-portal vein resection (SMPVR) may increase the rate of resection of PDCA, but the effect of this approach on improving long-term patient outcomes remains controversial. The present study investigated a case of PDAC in the pancreatic neck of a male patient. The patient underwent a TP, combined with SMPVR, for a margin-negative resection. Following an uneventful post-operative recovery, the patient received adjuvant chemoradiotherapy. The patient is currently alive at six years post-surgery, with a high quality of life. Given the clinical outcome of this patient, TP combined with SMPVR may provide PDAC patients with an opportunity for long-term survival. Therefore, patients with PDAC that is believed to be unresectable based on pre-operative assessment, may benefit from TP and SMPVR.

## Introduction

Pancreatic ductal adenocarcinoma (PDAC) is an aggressive cancer with limited therapeutic options. At present, surgical resection is the only potential curative treatment for PDAC. However, only 15–20% of patients with PDAC are eligible for lesion resection (1). Portal vein invasion is a significant factor that often precludes the resection of pancreatic tumors (2). In cases of portal vein infiltration, total resection of the pancreas and resection of the superior mesenteric-portal vein (SMPV) may increase the resectability. However, certain studies have suggested that SMPV involvement should be a contraindication for resection (3), as the combined resection approach may not improve overall survival. In the early 1950s, total pancreatectomy (TP) was introduced to prevent anastomosis-related complications (3), which contribute to the majority of hospital-related mortalities in PDAC patients. The high local recurrence rates following partial pancreaticoduodenectomies supported the notion that extended radical resections would improve long-term survival in cases of pancreatic malignancies. However, the use of TP to reduce hospital-related mortalities and improve patient outcomes remains controversial (4). Opponents of TP state that severe malabsorption and insulin-dependent diabetes mellitus are post-operative complications that may negatively affect quality of life following pancreatic resection (5). The present study investigated a case of PDAC in the pancreatic neck of a male patient. The patient underwent a TP, combined with an SMVP resection, for a margin-negative resection, and continues to survive six years after the surgery.

## Case report

On April 5, 2007, a 56-year-old male was admitted to Zhongshan Hospital (Fudan University, Shanghai, China) with a primary complaint of weight loss over the previous month. No abdominal or back pain was reported. Upon admission, the patient’s body weight was 58 kg, with a reported loss of 6 kg over the previous month. The patient denied alcohol and cigarette use. Jaundice was noticeable on the skin, but there was no sign of ascites. The patient was hospitalized with the suspicion of a malignant pancreatic neck tumor, without tenderness or a palpable mass in his abdomen, and without signs of diabetes mellitus.

**Figure 1:**
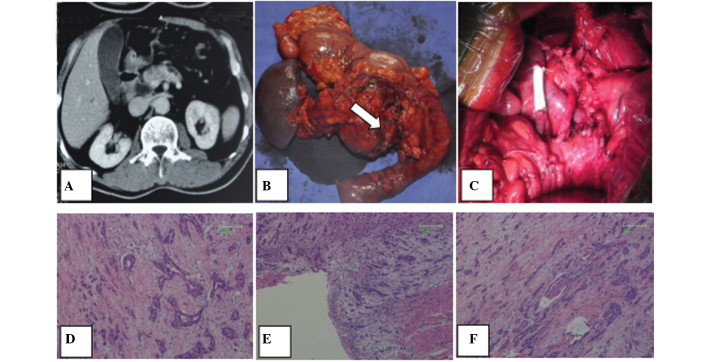
(A) Cross-sectional enhanced-abdominal computed tomography prior to surgery reavealing a low-attenuated mass in the pancreatic neck that was 3 cm in diameter and adhered to the portal vein. (B) Specimen from en bloc resection of the pancreas, spleen and superior mesenteric-portal vein (SMPV). The white arrow indicates the ~4-cm resected segment of the SMPV. (C) The surgical field post-surgery revealing the 4-cm vascular graft (GORE-TEX; diameter, 0.8 cm) used to carry out the vascular reconstruction. (D, E and F) Hematoxylin and eosin staining revealing a histological view of the resected pancreas (original magnification, ×100). (D) A moderately-differentiated invasive ductal adenocarcinoma (E) invaded the muscle layer of the portal vein wall and (F) peripancreatic nerve bundle.

The serum levels of total bilirubin and direct bilirubin were 82.1 μmol/l (normal range, 0.0–17.0 μmol/l) and 54.4 μmol/l (normal range, 0.0–9.0 μmol/l), respectively. In addition, the patient demonstrated elevated levels of several other laboratory markers, including 366 U/l aspartate aminotransferase (normal range, 0–75 U/l), 583 U/l alanine aminotransferase (normal range, 0–75 U/l), 649 U/l alkaline phosphatase (normal range, 15–115 U/l) and 333 U/l lactic dehydrogenase (normal range, 109–245 U/l). The amylase levels were 20 U/l (normal range, 0–200 U/l) and the carcinomatous biomarker, carbohydrate antigen 19-9 (CA19-9), was also elevated at 51 U/ml (reference range, 0–37 U/ml). The serum total protein, albumin and electrolytes levels were normal. The full blood counts were also normal. Computed tomography (CT) images revealed a low-attenuated mass that was 3 cm in diameter located in the neck of the pancreas. The tumor was adhered to the portal vein, but there was no evidence of distant metastasis (Fig. 1A). Based on these findings, the tumor was diagnosed as a pancreatic carcinoma involving the pancreatic neck, with SMPV invasion. Considering the conditions, it was decided that a TP, with a possible SMPV confluence resection, would be performed.

On April 12, 2007, the patient underwent a TP with splenectomy. Upon resection, it was revealed that the tumor had invaded the SMPV confluence. Therefore, a 4-cm segment of the SMPV (Fig. 1B) was resected for a margin-negative resection. The vascular reconstruction was performed using a 4-cm long vascular graft (GORE-TEX; diameter, 0.8 cm; Gore Medical, Newark, DE, USA; Fig. 1C). The total surgery time was five hours, and the total blood loss was 620 ml. The patient returned to an oral diet on the sixth post-operative day. The post-operative course was uneventful, and the patient was discharged on day 12 in a generally good condition. The final pathology report revealed a moderately-differentiated invasive ductal adenocarcinoma, with invasion to the muscle layer of the portal vein wall, the peripancreatic nerve perineurium and the anterior pancreatic capsule. The 12 lymph nodes examined were negative for any histological evidence of regional lymph node metastasis (Fig. 1D–F). Post-operatively, the patient received four cycles of gemcitabine (1,000 mg/m^2^d, days 1 and 8, every 3 weeks) as adjuvant chemotherapy. On July 7, 2007, an abdominal CT scan was performed for suspected lymph node metastasis in the retroperitoneal region. Subsequently, radiotherapy for the retroperitoneum in the surgically-resected region was performed. The patient attended follow-up examinations every six months. On April 7, 2012, a follow-up positron-emission tomography CT scan revealed a patent graft and no evidence of tumor recurrence. The tumor marker CA19-9 was also within normal limits at this follow-up. Currently, at the sixth post-operative year, the patient continues to survive.

The patient requires a regular dose of insulin (20 U/day), and at the last evaluation, the patient’s blood glucose was 108 mg/dl and the glycated hemoglobin was 6.2%. A course of digestive enzyme replacement (pancreatin enteric-coated capsules; Solvay, Brussels, Belgium) was initiated subsequent to the surgery, with a lipase dosage of 10,000 IU/kg/day. The patient has not complained of diarrhea with the digestive enzyme supplement, and maintains a stable body weight at 55 kg.

## Discussion

PDAC is the most common form of pancreatic neoplasm, accounting for >85% of pancreatic tumors (1). PDAC largely affects a patient’s quality of life and results in an extremely poor prognosis. Tumor resection is the only effective treatment (6). Despite the fact that surgery is the only potential curative intervention, only 15–20% of patients have resectable pancreatic tumors. Furthermore, only 20% of patients who undergo surgery experience survival at five-years post-surgery, resulting in an average five-year survival rate of 3–5% for all individuals diagnosed with PDAC (1)

Portal vein invasion is often a preclusive factor for surgery (2). Although resection of the portal vein and pancreas can be achieved safely, with little patient morbidity and mortality (7), the involvement of the SMPV is frequently considered a contraindication for resection. Certain studies have demonstrated limited effectiveness and low survival rates in cases of portal vein infiltration. A study by Allema *et al* (8) revealed that the rate of margin-negative resection was 15% in patients who had undergone SMPV resection. In addition, the study demonstrated that the overall one- and three-year survival rates were 59 and 16%, respectively. However, the mean survival-time was only 5.6 months in the margin-positive group. These findings emphasize the importance of negative pathological margins. Allema *et al* (8) also compared the survival rates between those patients with and without histological evidence of portal vein involvement. There was no significant difference identified between the patient groups, which suggested that SMPV invasion does not affect the survival rate of patients with PDAC (3). However, those patients with tumors that demonstrate invasion of the tunica intima are more likely to have a poorer outcome. Overall, no patients with tumor invasion to the tunica intima survive >6 months (9). Although invasion of the tunica media appears to be an important prognostic indicator, a diagnosis of tunica intima involvement prior to surgery is not possible.

TP was first introduced in 1943 by Rockey (10), and the technique was further described by Ross (11) in 1954. Over the past several decades, several clinical studies have supported the use of TP for the surgical management of pancreatic cancer (12–14). Proponents of this procedure argue that i) TP allows for a more extensive lymphadenectomy around the pancreas and leads to greater surgical ‘oncologic radicality’; and ii) TP decreases R1 and R2 resections, at least at the site of glandular transection (12). Data from the Mayo Clinic (13) has revealed the overall survival for patients with PDAC at one, two and three years post-TP to be 63, 43 and 34%, respectively. Reddy *et al* (14) reported that the five-year survival rate was 18.9% in 100 patients with PDAC who had undergone TP. These findings demonstrated that the long-term survival rate following TP was equivalent to survival rates after pancreaticoduodenectomy, and that TP should be performed when oncologically appropriate.

In the present case study, a patient with PDAC in the pancreatic neck underwent a TP and segmental resection of the SMPV. Post-operative pathological findings indicated that an invasive ductal adenocarcinoma had invaded the muscle layer of the portal-vein wall, the peripancreatic perineurium and the peripancreatic capsule. However, there was no evidence of regional lymph node metastasis, and resection margins, including the retroperitoneal margin, were negative. The patient currently reports a high quality of life and continues to survive six years after the surgery.

The primary aim of TP is to avoid a pancreatic-enteric anastomosis, as the principal cause of post-operative patient mortality following partial pancreatectomy is anastomotic leakage. Despite the intentions of this surgical approach, TP has not reduced hospital-associated mortalities (4), and there have been additional concerns regarding severe post-operative metabolic conditions that may arise with the complete removal of the pancreas (5). However, regardless of these initial concerns, morbidity and mortality following TP have decreased as a result of enhanced perioperative care and surgical techniques, and the presence of high-volume surgical centers (15). A number of centers have revealed perioperative mortality and morbidity rates of TP that are equivalent to those using the Whipple procedure (4). In addition, insulin-dependent diabetes and malabsorption following TP are better controlled with newer pharmacological interventions (15). In the present case study, the patient was administered insulin to control diabetes mellitus, and used a digestive enzyme to improve malabsorption following TP. The patient’s blood sugar and body weight currently remain stable and at normal levels.

Given the outcome of the present case, TP combined with SMPVR may provide PDAC patients the opportunity for improved long-term survival and a high quality of life. The TP and SMPV resection increases the surgical ‘oncologic radicality’ through extensive resection, and allows the surgeons to obtain negative margins. In conclusion, those patients with PDAC tumors typically believed to be unresectable based on pre-operative assessment may benefit from TP and SMPV resection.
